# Cri du chat syndrome and autism spectrum disorder: a case report

**DOI:** 10.1192/j.eurpsy.2023.849

**Published:** 2023-07-19

**Authors:** M. Raissouni, S. Benhammou, H. Kisra

**Affiliations:** Arrazi de Salé, SAlé

## Abstract

**Introduction:**

Cri du Chat syndrome (CdCS) is a genetic disorder resulting from a variable size deletion of the end of the short arm of chromosome 5 (5p), including a critical region located at p15.2. It represents one of the most frequent chromosomal deletions, with an incidence in the general population of 1/20,000 to 1/50,000.

**Objectives:**

Through this observation we update the scientific news of this rare syndrome and present an observation of a Cri du Chat syndrome confirmed by metaphasic karyotype (46,XY,del(5)(p13) de novo) with autism spectrum disorder.

**Methods:**

Description a case with cat cry syndrome seen in child psychiatry consultation in our institution

Discussion through articles published on pubmed, googlescholar and science direct

**Results:**

Typical features of CoCs present in the subject include intellectual disability, psychomotor acquisition delays, language delay, and dysmorphic features (e.g., wide and high nasal root, hypertelorism, and coarseness of features). Expected features of CoCs that are not present are: growth retardation, microcephaly, round facies, micrognathia, epicanthal folds and characteristic high-pitched cry. Behavioral features in this subject include symptoms of autism spectrum disorder.

**Conclusions:**

The deletion of the short arm of chromosome 5, when it includes a critical region located at p15.2, is responsible for a well-characterized syndrome, Cri-du-Chat disease, including a characteristic craniofacial dysmorphia that evolves with age, the mental handicap in the characteristic form is very severe. Visceral malformations are relatively rare and not very specific.

**Disclosure of Interest:**

None Declared

